# Soil physical properties of Austrian soils: Overview based on a comprehensive agricultural soil database

**DOI:** 10.1016/j.dib.2026.112702

**Published:** 2026-03-19

**Authors:** Florian Darmann, Monika Kumpan, Carmen Krammer, Thomas Weninger, Peter Strauss

**Affiliations:** Federal Agency for Water Management, Institute for Land and Water Management Research, Petzenkirchen, Austria

**Keywords:** Soil data, Laboratory measurements, Soil properties, Soil hydraulic parameters

## Abstract

Knowledge of soil physical characteristics is key for describing hydrological behavior of landscapes. Over the last few decades, several major data collections (e.g. UNSODA, HYPRES, EU-HYDI) have provided significant contributions to the field of hydrological modeling. However, these databases may exhibit limitations, when more detailed soil information is needed for particular regional applications. Our work presents a regionally focused, quality-controlled dataset on Austrian soils. It provides soil data from studies on various land use types and soil management systems, obtained during long-term monitoring and short-term project work. The dataset contains detailed information collected over the last 30 years on 2203 soil profiles in total, representing a variety of landscapes and soil types from Austria’s small-structured and diverse geography. Both disturbed and undisturbed samples are included, to obtain a wide spectrum of basic and hydraulic soil properties. The disturbed samples include measurements of soil texture (*n* = 6406), determined by sieving and sedimentation analysis, as well as organic carbon content using two different methods (*n* = 3671 and *n* = 2862). The undisturbed samples were used to determine bulk density (*n* = 5768) and to analyze soil hydraulic properties (SHPs). SHPs were determined with different methods, depending on the period of sampling. Older samples were analyzed using the pressure plate apparatus (*n* = 2403), while more recent samples were analyzed using the HYPROP device that applies the evaporation method in combination with the dewpoint method. A unique profile ID is assigned to each soil profile, enabling links between the different subsets. The metadata includes the coordinates of the sampling locations, the sampling data and the number of sampled depths per profile. Reclassified land use information is available for all profiles, based on CORINE 2018 land cover. The dataset provides detailed, long-term soil information from projects across Austria over a period of more than 30 years. It may be used for descriptive statistics, as well as for identification of soil hydrological parameters on a regional or national scale.

**Table:** Specifications Table

Subject	Earth & Environmental Sciences
Specific subject area	Soil physical, chemical, and hydraulic properties of Austrian soils
Type of data	Table, Raw, Analyzed, Filtered
Data collection	Field sampling campaigns and long-term monitoring sites; laboratory analyses using international standards
Data source location	Country: Austria; sampling sites distributed across the main agricultural regions, and selected forest and alpine areas
Data accessibility	Repository name: Zenodo: Data identification number: 10.5281/zenodo.17801707 Direct URL to data: https://doi.org/10.5281/zenodo.17801707
Related research article	none

## 1 Value of the Data

• This dataset provides comprehensive, long-term soil data on Austrian soils, collected over a period of more than 30 years.
• By combining data from project-based sampling and long-term monitoring, it provides information on the physical, chemical, and hydraulic properties of Austrian soils.
• The dataset is ready to use for statistical analysis, hydrological modeling, or for the development of regional pedotransfer functions.
• These data could be useful for researchers in soil science, hydrology, and environmental sciences, as well as for stakeholders and engineering agencies working in agricultural or water-resource management

## 2 Background

Describing ecological, agricultural and water-related processes requires an understanding of soil characteristics. Therefore, in order to evaluate the influence of specific soil properties on hydrological functions, it is essential to have a thorough understanding of those properties. These properties include soil texture, bulk density and organic carbon content, as well as soil hydraulic properties (SHPs) such as water retention capacity and hydraulic conductivity. Information on the SHPs can be used for further modeling of landscape hydrology.

Multiple datasets containing soil physical data have been published and made be available to researchers during the last few decades, including the Unsaturated Soil Hydraulic Database (UNSODA [1]), the Hydraulic Properties of European Soils Database (HYPRES [2]) and the European Hydropedological Data Inventory (EU-HYDI [3]). Other relevant datasets have been provided by Schindler and Müller [4], Hohenbrink et al. [5], and Gupta et al. [6]. The datasets contain information about SHPs together with fundamental soil properties like soil texture or organic carbon content, but they vary in terms of data scope, spatial coverage, and data quality. They contain regional information and have also been used to derive pedotransfer functions (PTF) for the respective region. The UNSODA dataset was utilized to develop the PTF tool ROSETTA [7], while the EU-HYDI dataset was the basis for the EU-PTF (euptf [8,9]).

This work presents a dataset, which contains Austrian soil data, from a variety of Austrian landscapes and soil types, enabling a thorough description of the spatial variability of their physical, chemical and hydraulic properties.

## 3 Data description

The dataset is structured into multiple subsets:

(a) metadata (meta.csv)
(b) soil physical properties (physical.csv)
(c) soil chemical properties (chemical.csv)
(d) various soil properties (various.csv)
(e) soil hydraulic properties with retention and conductivity characteristics included (ret.csv; cond.csv)

**Fig. 1:**
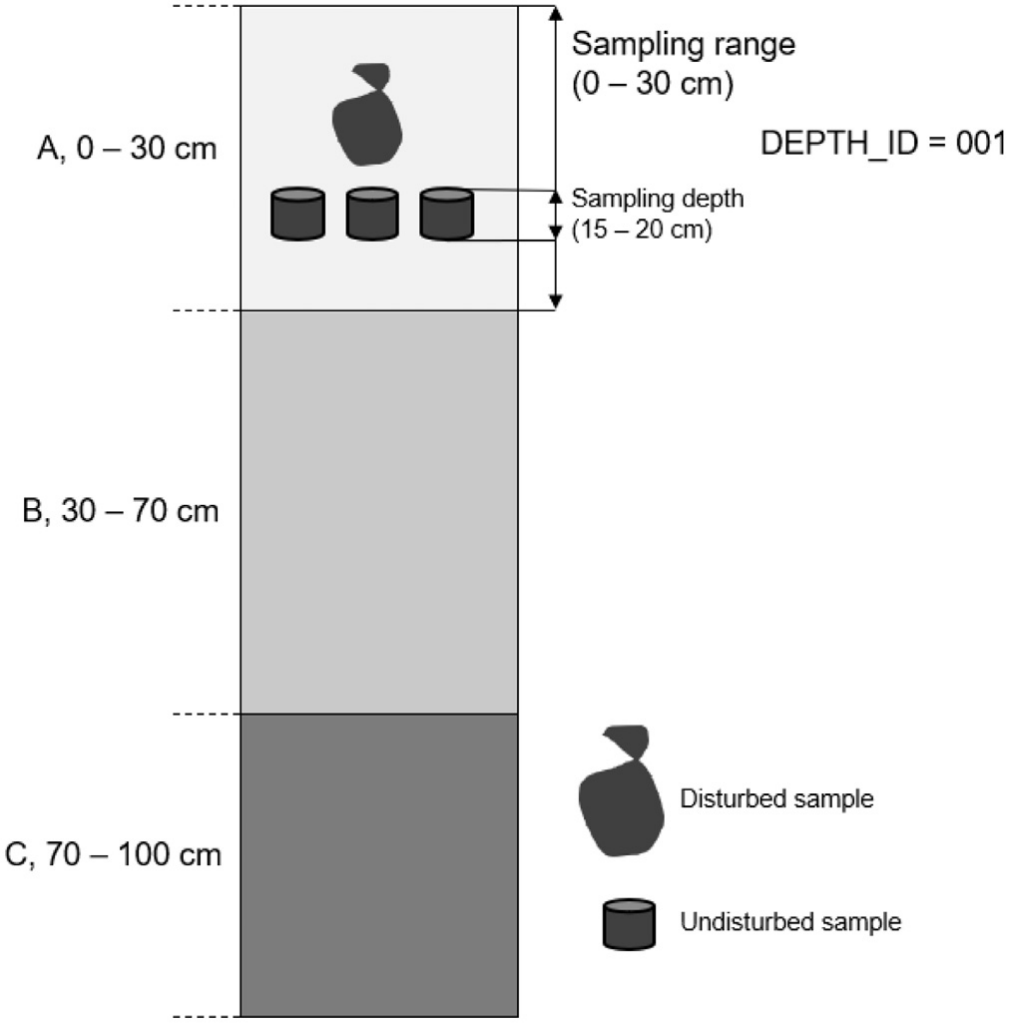
Schematic illustration of the hierarchical sampling structure within a soil profile. Disturbed samples were collected across defined sampling ranges (e.g. soil horizons or fixed depth intervals such as 0–30 cm). Undisturbed samples were taken at specific sampling depths within these ranges (e.g. 15–20 cm). Both sample types were assigned a shared Depth_ID to link measurements from the same depth interval.

A distinctive, unique ID (IKT-**) has been allocated to each profile to establish a connection across the subsets. Within each profile, samples were taken at various depths. Disturbed samples were collected according to soil horizons or predefined depth intervals. Undisturbed samples were taken at specific sampling depths within these intervals. Each sampling interval was assigned a Depth_ID to link the corresponding disturbed and undisturbed samples across the subsets, allowing multiple analyses of the soil profiles' physical, chemical and hydraulic properties. Fig. 1 illustrates the hierarchical sampling structure within a soil profile.

Profiles with identical coordinates that were resampled at a later date were given their own profile IDs. As a result, they do not necessarily have the same assignment of sample ID according to the sampling range and number of samples. They are not always directly comparable over the years. The number of samples and sampling depth per profile vary depending on the research questions of the respective project. The distinctive profile ID-system allows straightforward integration of the various tables and enables adaptable searching by location, depth, and parameter availability.

### 3.1 Meta table

The Meta table provides a structured overview of the general information for each profile. It contains comprehensive data on 2203 profiles with varying depths, typically extending up to a maximum depth of 200 cm, though some exceptions in groundwater monitoring range up to deeper levels. Each profile entry includes:

(a) the unique profile ID and the number of depths
(b) GPS coordinates (WGS84)
(c) sampling time, where available
(d) land use based on CORINE land cover data
(e) data availability flags (TRUE/FALSE) for the physical, chemical, various, and hydraulic data categories.

These flags allow profiles to be filtered quickly according to data availability. The coordinates are available in WGS84 format for every profile, sites without coordinates are not included. Profile IDs were assigned in sequential order over time, with lower numbers referring to earlier sampling campaigns. These IDs do not represent any spatial or categorical structure.

Information on land use is available for all profiles, based on CORINE land cover data with a resolution of 100×100 m [10]. The original 44 land use classes are included (code 118), as well as a reclassified version summarized into seven categories: urban/infrastructure; agriculture; forest; low-vegetated areas; alpine areas; wetlands; and water bodies.

2149 profiles contain at least one entry of soil physical properties, while 1369 profiles include information on the chemical properties of the soil. The dataset includes 2413 measurements with a pressure plate apparatus, and 1281 samples from 167 sites measured with a HYPROP device. All 167 sites also have analyses concerning the basic soil properties. Table 1 summarizes selected soil properties and their respective value ranges within the dataset. The number of samples (n) includes all available measurements for each parameter and may therefore comprise values obtained with different analytical methods (e.g. soil organic carbon determined by wet oxidation or dry combustion, and water retention measured using a pressure plate apparatus or a HYPROP device).

**Table 1:** Summary statistics of selected soil properties included in the dataset. **The number of samples (n), minimum (Min), maximum (Max), mean, median, and the 5% and 95% percentiles (Perc) are shown for each parameter to provide an overview of the range and variability of the dataset.**

	**n**	**Min**	**Max**	**Mean**	**Median**	**5% Perc**	**95% Perc**
Number of profiles	2203						
Bulk density (g cm^−^³)	5768	0.1	2.2	1.4	1.4	1.0	1.7
Soil organic carbon content (%)	3671	0.0	44.7	1.7	1.6	0.2	3.0
Soil organic matter (%)	2862	0.0	26.0	1.7	1.7	0.2	3.4
pH (-)	2587	3.2	8.7	6.9	7.3	5.0	7.9
Saturated hydraulic conductivity (cm d^−1^)	4213						
**Particle size distribution**							
Sand (%)	6406	0.5	98.3	24.1	16.6	3.9	71.3
Silt (%)	6406	1.2	87.6	53.9	57.7	21.1	74.2
Clay (%)	6406	0.0	77.8	22.0	22.5	4.0	36.7
**Water retention curve by method**							
Pressure plate apparatus	2413						
HYPROP	1281						

### 3.2 Basic soil properties

**Fig. 2:**
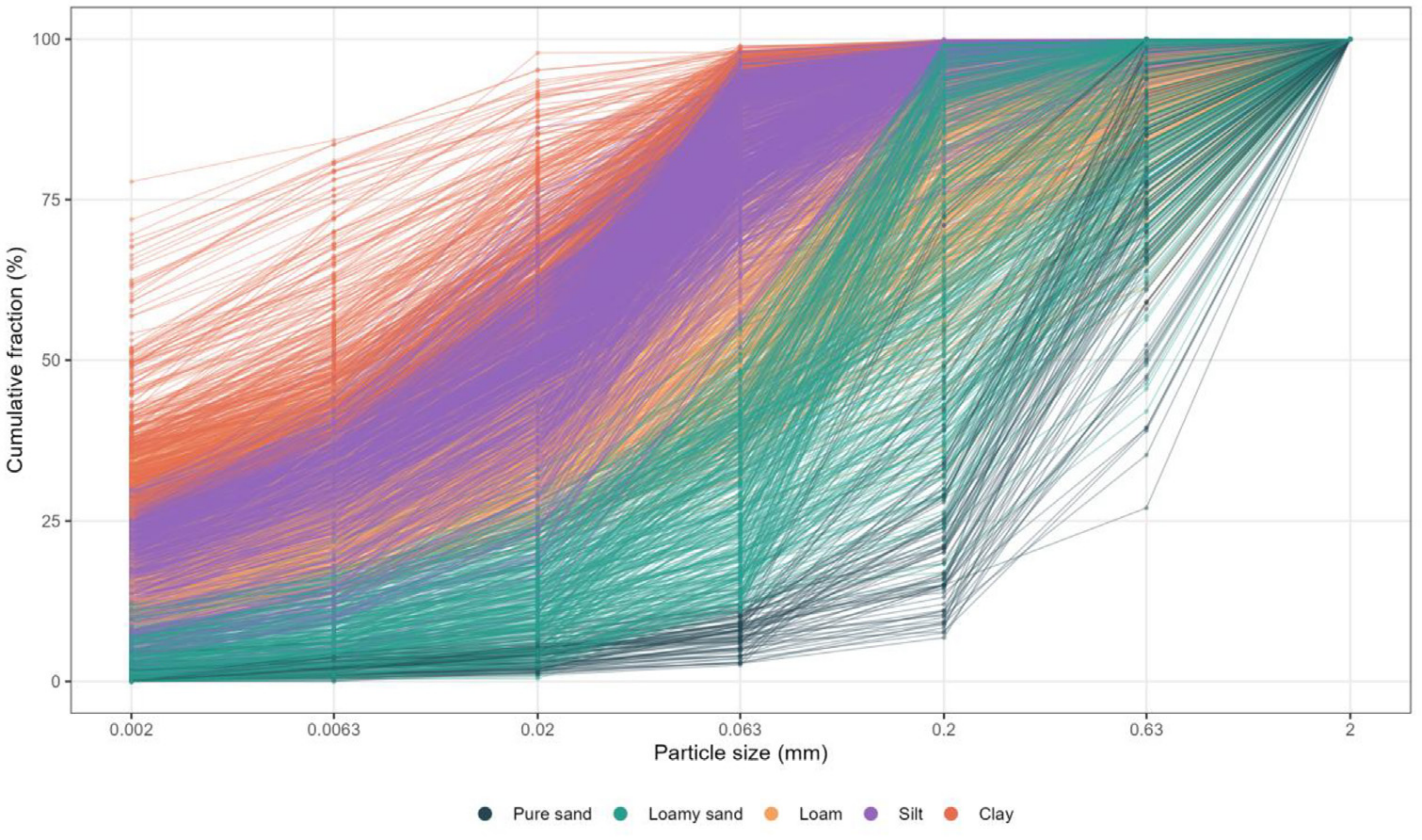
Cumulative particle size distribution for the main textural classes sand, loamy sand, loam, silt and clay.

The included soils cover a broad spectrum of textural classes, as defined by the German classification system. The dataset consists of 6404 particle size distribution measurements, with sand content of up to 98.3%, silt content of up to 87.6% and clay content of up to 77.8%. Most of the samples are classified as silty loam (18.8% of the samples), a texture class that commonly occurs in Austrian agricultural soils [11]. For the upcoming analysis, the texture classes were reclassified into the main textural categories: sand (*n* = 653; 10.2%), loam (*n* = 1298; 20.3%), silt (*n* = 2766; 43.2%) and clay (*n* = 1689; 26.4%), according to the German classification system. Soils belonging to the sand fraction show low water retention capacity and high saturated conductivity. These samples are subdivided into pure sand and loamy sand. Fig. 2 illustrates the cumulative particle size distribution, classified into the five main textural classes.

**Fig. 3:**
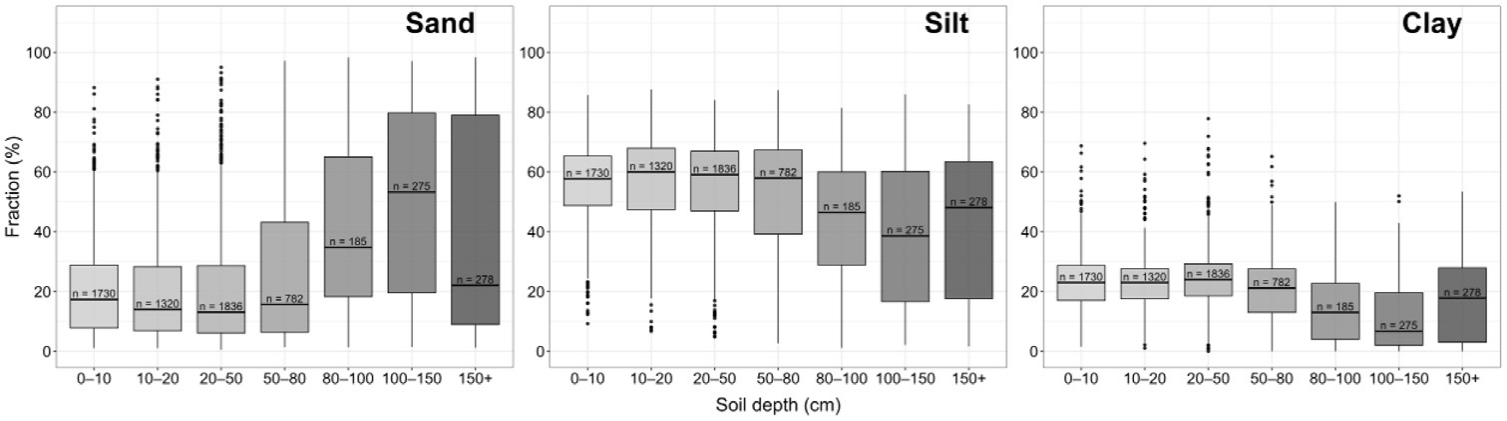
Soil texture fractions (sand, silt, clay) for individual depth intervals (cm), *n* = number of observations for each depth interval.

Fig. 3 shows the variation of sand-, silt-, and clay content with soil depth. The sand content increases with depth, particularly beyond 80 cm. In contrast, the silt content remains constant up to 50 cm and then decreases consistently. Clay content shows a comparable pattern with a moderate decrease below 50 cm. The number of samples varies between 1730 samples in topsoil (0 – 10 cm) to 278 samples in the deepest depth interval (> 150 cm).

**Fig. 4:**
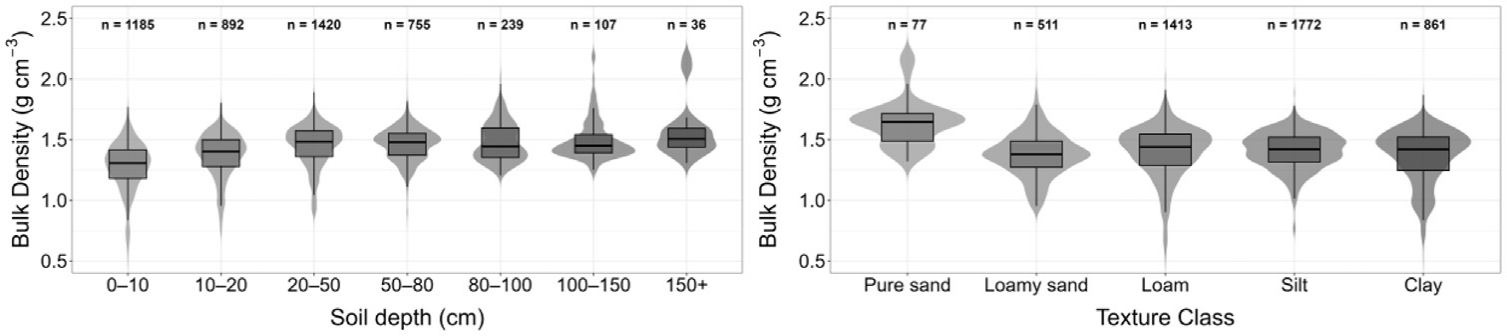
Distribution of bulk density depending on soil depth (cm, left) and main textural classes (right). Boxplots illustrate the median and interquartile range, violin plots show the data density. *n* = number of samples for each category.

The bulk density measurements in the dataset range from 0.1 g cm^−3^ to 2.2 g cm^−3^, with a median value of 1.4 g cm^−3^ (see Table 1). The distribution of bulk density by depth and main textural class is shown in Fig. 4. A consistent increase with depth can be observed. Pure sands show higher bulk densities than clay and silt soils, for which bulk density remains constant.

**Fig. 5:**
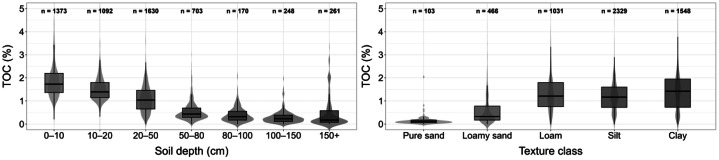
Distribution of organic carbon depending on soil depth (cm, left) and main textural classes (right). Boxplots illustrate the median and interquartile range, violin plots show the data density. The number of samples (n) for each category is displayed at the top.

The topsoil layer (0 – 10 cm) contains the highest organic carbon levels which reach 1.7% on average. The organic carbon levels decrease steadily as the soil depth increases beyond 100 cm until they reach below 0.5%. Fine-structured soils (e.g. loam, silt and clay) contain the highest organic carbon levels. The results are displayed in Fig. 5.

### 3.3 Soil hydraulic properties

**Fig. 6:**
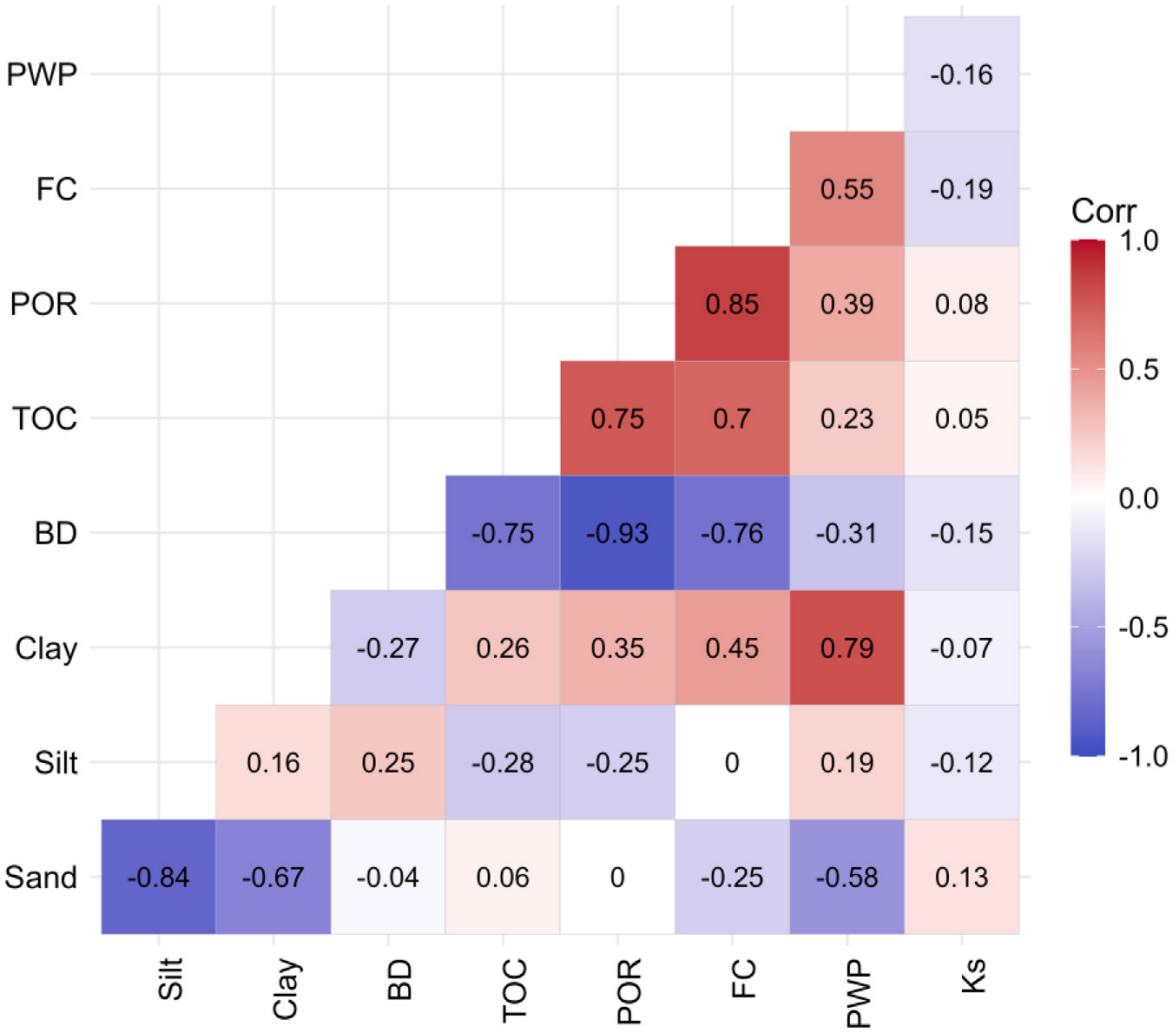
Correlation matrix with pairwise Pearson coefficients for selected soil properties: PWP = water content at pF 4.2, FC = water content at pF 1.8, POR = saturated water content, TOC = total organic carbon, BD = bulk density; Ks = saturated hydraulic conductivity. Blue squares indicate negative correlation, red squares indicate positive correlation between two parameters.

Fig. 6 shows a correlation matrix with pairwise Pearson correlation coefficients for selected soil properties, such as bulk density (BD), organic carbon content (TOC), soil texture (sand, silt, and clay), and soil hydraulic properties derived from HYPROP measurements, including saturated water content (POR), water content at pF 1.8 (FC), water content at pF 4.2 (PWP), and saturated hydraulic conductivity (Ks).

A negative correlation between sand content and the permanent wilting point (*r* = −0.58) is observed. Clay content correlates positive with permanent wilting point (*r* = 0.79) as well as organic carbon content with saturated water content (*r* = 0.75) and field capacity (*r* = 0.7). For the saturated hydraulic conductivity, only weak correlations (e.g. *r* = 0.13 with sand content) without any trends are identified for this dataset. Strong negative correlations are observed between bulk density and saturated water content (*r* = −0.93), as well as bulk density and field capacity (*r* = −0.76). Additionally, bulk density shows a negative correlation with organic carbon content (*r* = −0.75).

**Fig. 7:**
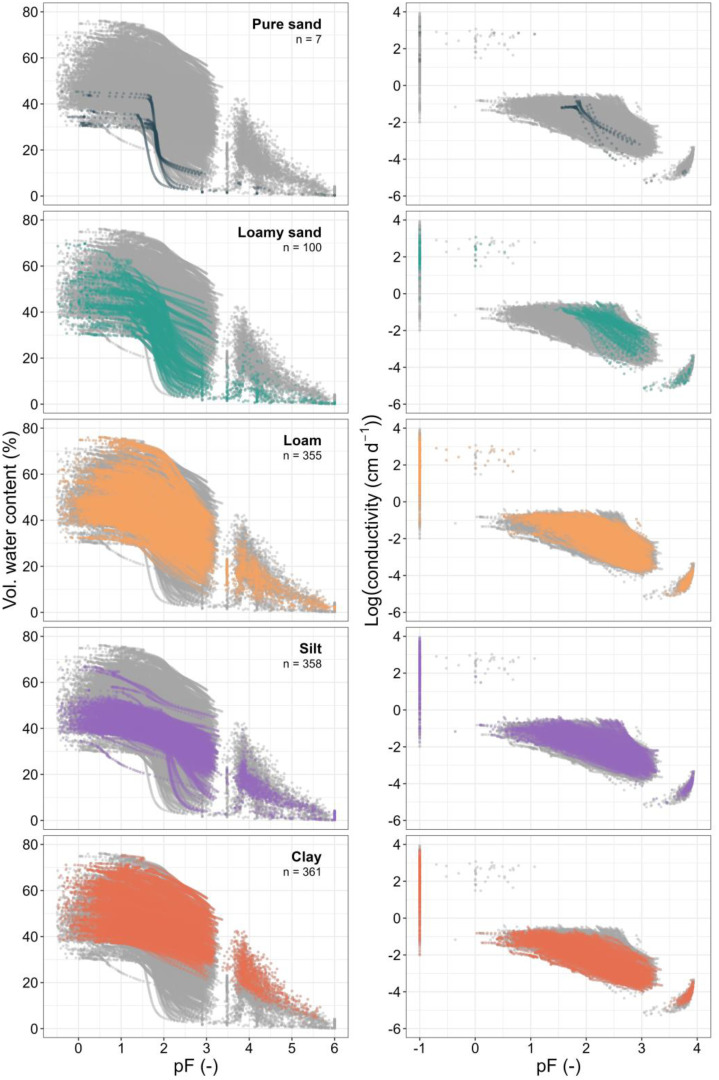
Soil water retention (left) and unsaturated hydraulic conductivity (right), categorized by the main texture classes (*n* = 1181; number of samples). Volumetric water content and log-transformed hydraulic conductivity are shown on the y-axis as functions of matric potential. A combination of different methods is used. Grey background points represent the complete dataset and illustrate the overall variability across all samples. Color-coded points in subplots highlight the respective main texture classes: pure sand, sandy loam, loam, silt and clay.

Fig. 7 illustrates the results for soil water retention (left) and unsaturated hydraulic conductivity (right), categorized by the main texture classes (*n* = 1181). Volumetric water content and log-transformed hydraulic conductivity are shown on the y-axis as functions of matric potential. Due to the evaporation method used, most of the data points are found between pF 0 and pF 3.5. All water retention samples include an additional data point between pF 3.5 and 4.0, representing the air entry pressure point. Additional measurements are taken at >pF4, relating to the dew point method (WP4C device). The number of WP4C measurements varied between one and three, but was usually set to three for each sample. The dots displayed at pF −1 for hydraulic conductivity show the results from KSAT measurements. Silt and clay soils retain more water across the pF range, while sandy soils have a lower water-holding capacity with rapidly reduced water content beyond pF 2.5.

**Fig. 8:**
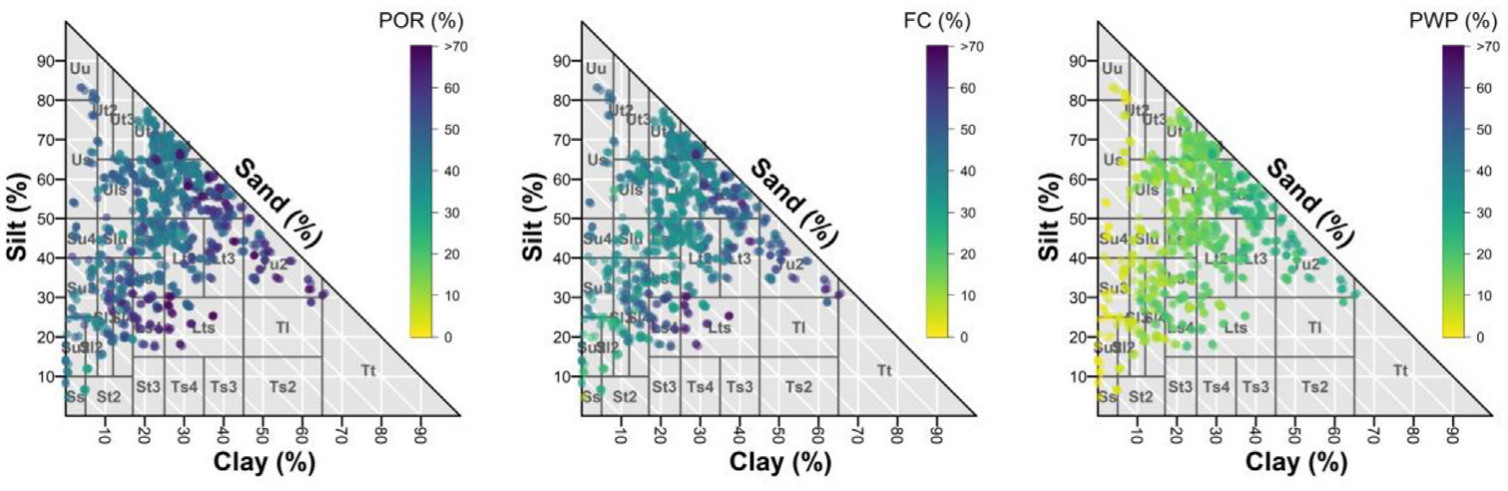
Measured volumetric water contents (%) derived from HYPROP measurements at saturation (POR; left), at pF 1.8 (FC; middle), and at pF 4.2 (PWP; right), displayed on a soil texture triangle. Only samples with available paired soil texture information (sand, silt, clay) were included (*n* = 1181).

Fig. 8 shows the relationship between soil texture and hydraulic characteristics by displaying the saturated water content (POR) and the water content at specific pF values across the texture triangle. Finer-textured soils (e.g. silty and clay-rich soils such as Tu2 and Lt3) show higher water retention values than coarser-structured soils. Sand-dominated soils, on the other hand, have lower saturated water content ( <40%), lower field capacity, and permanent wilting point values ( <10%).

### 3.4 Pedoclimatic characteristics

**Fig. 9:**
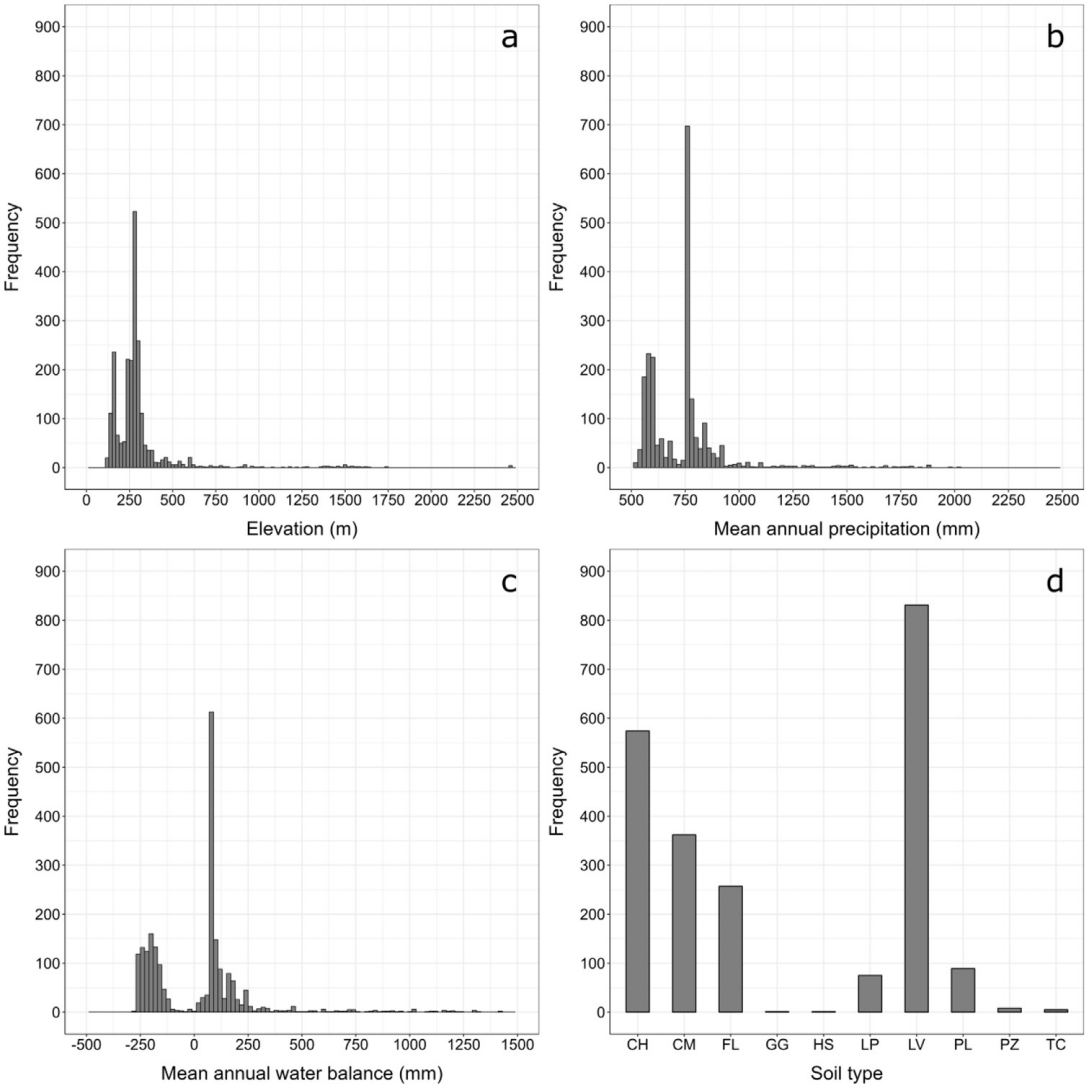
Pedoclimatic distribution of the sites included in the dataset for a) elevation (m), b) mean annual precipitation (mm), c) mean annual water balance (mm), and d) soil types. Soil types are classified based on the World Reference Base for Soil Resources (IUSS Working Group WRB, 2022), abbreviated as: CH = Chernozems, CM = Cambisols, FL = Fluvisols, GG = Glaciers, HS = Histosols, LP = Leptosols, LV = Luvisols, PL = Planosols, PZ = Podzols, TC = Technosols. The histograms show the frequency distribution of climatic and pedological characteristics of all sampling locations.

We analyzed the distribution of selected environmental properties, such as elevation [12], mean annual precipitation [13], mean annual water balance [13] and soil type classification [14]. The results in Fig. 9 illustrates the frequency distribution across the sampling locations. The majority of the sites are located at an altitude between 100 and 500 m, with mean annual precipitation values between 550 and 900 mm. The water balance of the sites varies, with some sites showing a slightly negative yearly balance. The main soil types included in the dataset are Luvisols, followed by Chernozems, Cambisols and Fluvisols. These soil types are commonly found in lowland areas, where agriculture is the dominant land use. Fewer sites are located at higher elevations or in drier regions.

## 4 Experimental design, materials and methods

### 4.1 Data source and sampling campaigns

**Fig. 10:**
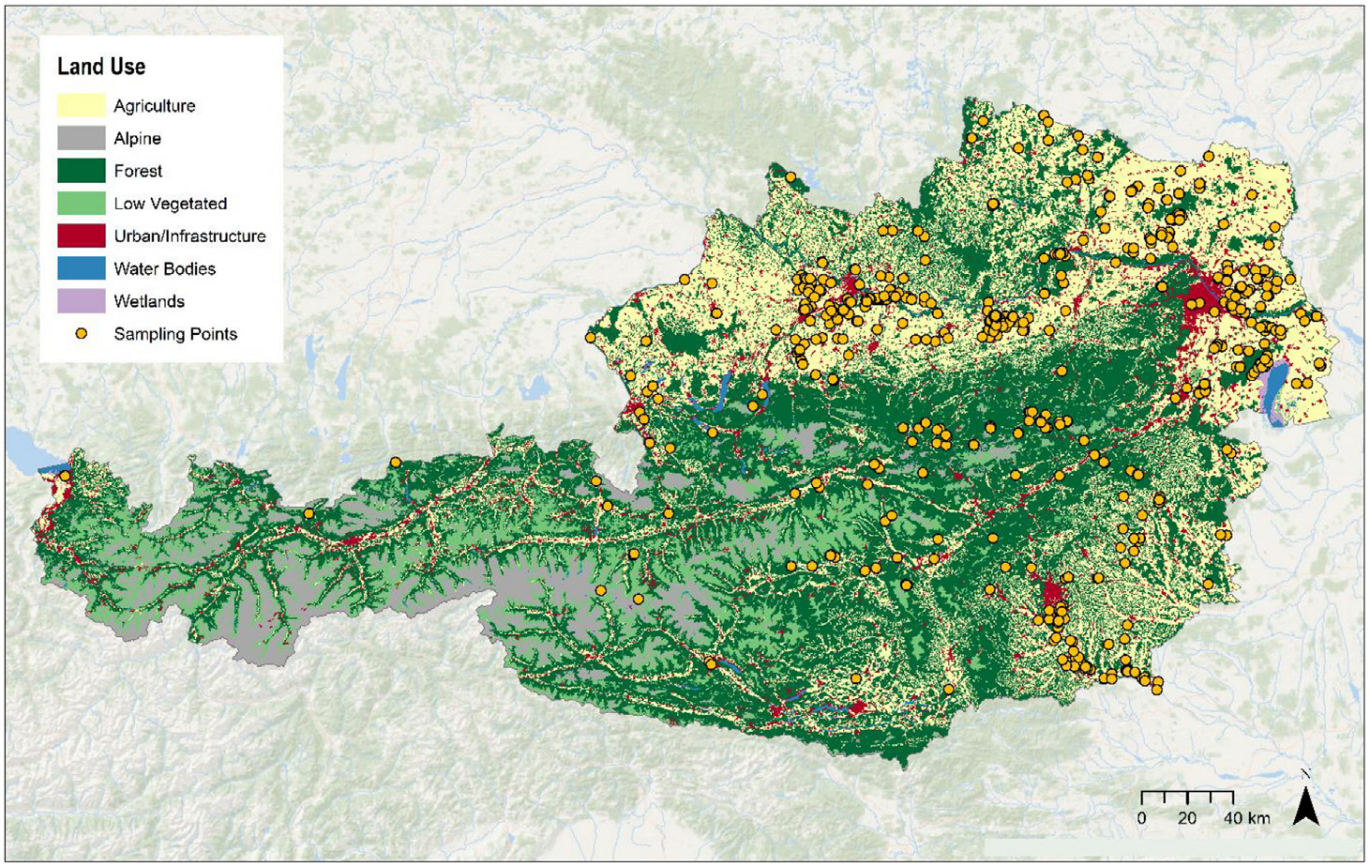
Location of sampling points across Austria. Reclassified land use map based on CORINE land cover with a resolution of 100×100 m (Umweltbundesamt, 2018).

The dataset, which is hosted by the Federal Agency for Water Management, includes soil data gathered from 1992 to 2024 in order to answer specific research questions. It represents soil properties from agricultural areas with varying soil types and landscapes in Austria. Most of the sampling sites are located in Lower and Upper Austria. Additional sites are situated in Burgenland, Salzburg and Styria, while a smaller number of samples were collected in Carinthia, Tyrol and Vorarlberg. Fig. 10 provides an overview of all sampling sites in Austria.

### 4.2 Sample processing

The dataset is primarily organized into soil profiles, consisting of disturbed and undisturbed samples. The undisturbed samples were taken using steel sampling cores (200 or 250 cm³) at a specific depth, and were used to determine bulk density, water retention curves, and unsaturated hydraulic conductivity. The disturbed samples were taken from the area immediately surrounding the cores. They were aligned either to soil horizons or to specific depths, and cover therefore a wider depth range than the undisturbed samples. These samples were used to estimate the particle size distribution, organic matter content, and chemical parameters such as pH value and electrical conductivity. All sampling points were georeferenced. Sampling sites without coordinates are not included.

### 4.3 Laboratory measurements

The samples were analyzed in accordance with national and international standards, that were state of the art at the time of analysis. To ensure comparability and consistent interpretation, the dataset only contains samples that were analyzed with established methods. Due to the long period of data collection, some of the used methods have been modified, since they are subject to further development. An overview of the applied methods is shown in Table 2, the following section summarizes the main laboratory procedures.

**Table 2:** Analytical methods for determining soil parameters and their standards. **Abbreviations are provided in parentheses. Explanation of column headers: REF_ISO = reference to the according ISO-standard, REF_ÖNORM = reference to the according Austrian standard. References to the notes are given in the manuscript text below.**

Parameter	Method	REF_ISO	REF_ÖNORM	Notes
Bulk density (BD)	Undisturbed soil cores, in steel cylinders (200 mL), oven-dried at 105 °C (48 h)	ISO 11272	L 1068	–
Particle size distribution (PSD)	Sieving and sedimentation of fine earth (<2 mm): fraction borders: 2 mm ≥ Sand > 0.063 mm ≥ Silt > 0.002 mm ≥ Clay	ISO 17892-4	L 1061–2	–
	Sieving of coarse fraction (>2 mm) after aggregate disruption	ISO 17892-4	L 1061–1	–
Soil organic matter (SOM)	Loss on ignition	–	L 1079	–
Soil organic carbon (SOC)	Dry combustion	ISO 10694	L 1080	–
	Wet oxidation	–	L 1081	–
Carbonates (CaCO₃)	Scheibler method	ISO 10693	L 1084	–
pH	Suspension in CaCl₂ solution	ISO 10390	L 1083	–
Water retention curve (WRC)	Pressure plate apparatus	ISO 11274	L 1063	–
	Evaporation method	ISO 11275	–	HYPROP
	Dewpoint method	–	–	WP4C
Saturated hydraulic conductivity (Ks)	Constant or falling head method	ISO 17892-11	L 1065	KSAT
Unsaturated hydraulic conductivity (Ku)	Evaporation method	ISO 11275	–	HYPROP

#### 4.3.1 Physical properties

The particle size distribution of the disturbed samples was determined by a combination of sieving for the sand fraction and sedimentation for the silt fraction and the clay content. The texture classes were defined in accordance with the German soil classification system with fraction borders from <0.002 mm for clay, <0.063 mm for silt and up to 2 mm for fine sand [11]. In order to identify coarse fragments, particles that do not pass through a 2 mm sieve are weighed. Bulk density was measured for undisturbed samples, which are taken with the mentioned steel cylinders with known volume. The samples were oven-dried at 105 °C for 48 h and the bulk density is calculated from weight and the volume of the cores.

#### 4.3.2 Chemical properties

Soil organic matter is defined by loss of ignition, while the determination of organic carbon is conducted via wet oxidation. Furthermore, the measurement of organic carbon is completed through the use of dry combustion, without the consideration of carbonates. As a result of the fact that for certain profiles either SOM or OC is available, the missing data points were calculated using a specific factor (soil organic matter = 1724 * organic carbon). pH was measured in a CaCl2 solution.

#### 4.3.3 Hydraulic properties

Several methods were employed to assess the soil water retention characteristics of the samples. Earlier samples were analyzed using a pressure plate apparatus to measure water content at specific matric potentials. More recent samples were analyzed with the HYPROP device (Hydraulic Property Analyzer, METER Group, AG, Germany), to determine water retention curves using the simplified evaporation methods [15,16]. Furthermore, the full range of matric potentials was covered employing the dew point method using the WP4C device [17].

The HYPROP system also enabled the unsaturated hydraulic conductivity of the same samples to be determined based on Darcy's law. Additional K measurements were gained from adaptations of Wind’s evaporation method [18], or Schindler’s approach [15]. The saturated hydraulic conductivity of the undisturbed samples was determined using either the constant or falling head methods, both of which are implemented in the KSAT device.

## Limitations

A few limitations should be noted for this dataset. The objectives of the sampling campaigns represented in this dataset varied. Depending on the specific research questions, the dataset includes studies on different land use and soil management strategies, long-term monitoring studies and method development. While each site contains the relevant analyses required for the respective project, these vary depending on the research question. As the projects mainly focus on agricultural issues, an uneven spatial distribution of sampling sites can be observed. As the main agricultural sites are located in Lower and Upper Austria, soils there were sampled frequently, leading to overrepresentation of certain types of soil. Although additional information on land use and crop sequences is not available, this dataset provides soil data across diverse Austrian landscapes. Furthermore, analysis methods have changed over the years, resulting in differences between some measurements.

## Future Applications

The new BAW database presents soil data from all over Austria, including regions previously not available in other datasets. While a subset of this dataset has been used for PTF development in previous studies [9,19] the dataset in its current state (as of 2024) was not part of any model development. Due to its spatial coverage, the dataset can support regional soil mapping applications. Combined with other datasets from other countries, it could be part of an international data collection.

## Data Availability

The dataset can be downloaded from Zenodo (zenodo.org), an open source data repository hosted by research institutions and supported by federal institutions of Germany and the European Union. The dataset is available at https://doi.org/10.5281/zenodo.17801707 [20]. Results from new measurement campaigns will be added frequently to the same location (DOI) and data structure. The statistics and analyses below describe the dataset as of September 30, 2025.

## Ethics Statement

This work complies with the ethical requirements for publication, since it does not involve human subjects, animal experiments, or any data collected from social media platforms.

## CRediT Author Statement

**Florian Darmann**: Conceptualization; Data curation; Formal analysis; Writing – Original Draft; Writing – Review & Editing; Visualization. **Monika Kumpan:** Investigation; Supervision. **Carmen Krammer:** Data curation. **Thomas Weninger:** Conceptualization; Supervision; Writing – Original Draft; Writing – Review & Editing. **Peter Strauss:** Supervision; Writing – review & editing.

## Declaration of generative AI and AI-assisted technologies in the writing process

During the preparation of this work the author(s) used Grammarly in order to improve the language and readability. After using this tool/service, the author(s) reviewed and edited the content as needed and take(s) full responsibility for the content of the publication.
